# Investigating Visual Monitoring of the Scrotum as a Supplementary Tool for Boar Semen Quality Evaluation

**DOI:** 10.3390/vetsci10010009

**Published:** 2022-12-24

**Authors:** Vasiliki Stravogianni, Theodoros Samaras, Constantin M. Boscos, Athina Basioura, Ioannis Markakis, Ioannis A. Tsakmakidis

**Affiliations:** 1 School of Veterinary Medicine, Faculty of Health Sciences, Aristotle University of Thessaloniki, 54627 Thessaloniki, Greece; 2 School of Physics, Faculty of Sciences, Aristotle University of Thessaloniki, 54124 Thessaloniki, Greece; 3 Department of Agriculture, School of Agricultural Sciences, University of Western Macedonia, 53100 Florina, Greece

**Keywords:** sperm analysis, scrotum, boar, video recording, behavior, prognosis, fertility

## Abstract

**Simple Summary:**

Laboratory tests and the recording of the artificial insemination (AI) field outcomes are used to predict boar fertility. In the context of the continuous efforts to improve AI results and produce a reliable prognosis of the male fertilization capacity, new techniques have also been introduced based on the use of sensors, image recording, behavioral studies, etc. In this direction, the possibility to associate scrotal contractions features, observed during the semen collection procedure, with important boar sperm parameters was studied.

**Abstract:**

Farm animals behavior research uses video cameras, mainly for visual observation and recording. The purpose of this feasibility study was to enrich the predictable methods of boar semen production capacity by correlating sperm variables with the scrotal contractions (SC) frequency and intensity. A video camera was used to record the reaction of the scrotum during ejaculation. The respective collected ejaculates were evaluated and semen parameters, such as viability, morphology, membranes functional integrity and kinematics, were determined. The camera recorded the scrotal contractions/relaxations and the video was handled by the Image Processing Toolbox of Matlab (Mathworks Inc., Natick, MA, USA). The SC intensity was verified as a percentage change in the scrotum size among the video frames of maximum contraction and relaxation. The archived data from the frames were analyzed statistically, using a linear mixed effects model that involved sperm assessed parameters. Correlations of the SC intensity with the average path velocity, VAP (R^2^ = 0.591, *p* = 0.043) and with the percentage of the cytoplasmic droplets (R^2^ = 0.509, *p* = 0.036) were noticed. Previous studies reported the positive correlation of VAP with the number of live-born piglets. In conclusion, video monitoring of the boar scrotal function during ejaculation is useful, but more research is needed to establish its appropriateness as a supplementary method for the prognosis of boar ability to produce high-quality semen.

## 1. Introduction

Nowadays, boar liquid semen is used globally to inseminate sows, supporting pig farm productivity. Considerable attention to male reproductive management had developed to meet the needs of the market. High number of boars are housed in specialized artificial insemination (AI) centers for the production and distribution of semen. Alternative, they are kept in pig farms, producing and providing high-quality semen to fertilize the females. From that point of view, boar semen production capacity is a primary supporting factor for the efficacy of AI, while the boar is a critical unit for a financially successful pig farm. Many laboratory techniques exist to evaluate and ensure boar spermatozoa fertilizing ability. In aiming to find a reliable prognostic method of assessing boar field fertility, laboratory tests are considered good, but are not enough. In recent years, different strategies have been used to approach in vivo sperm fertilization capacity. Seminal plasma markers of fertility have been identified, while analytical molecular biochemistry investigates sperm genomics, proteomics, and epigenetics [1,2,3,4]. Biomedical sensors and technical equipment for the automated monitoring of the male animals have been involved in these prognostic efforts [5], and video cameras use is more and more involved in animal behavior studies. Waters et al. [6] reported that visual observation of sheep lying and contractions could be utilized as an alert to the parturition progress. In pigs and buffaloes, camera monitoring became an essential tool to evaluate health status and detect on time common visual signs of respiratory and digestive diseases [7,8]. However, research about the correlation of boar semen quality characteristics with the data obtained by visual cameras during the ejaculation process was not found.

Furthermore, the boar is a male animal with time-consuming and completely different behavior during ejaculation compared to other mammals. The lengthy mounting of a boar on a dummy allows thorough visual recording of its visible genital system. On the other hand, the supporting role of the scrotum on the normal spermatogenesis by testicular thermoregulation is well known [9], while any factor affecting testis reflects semen quality. Artificially induced heat shock environmental conditions, as well as ultrasonographical and thermographic techniques, have been used to study boar scrotum function and sperm susceptibility [10,11]. Considering that different assays must be applied to enrich semen assessing and advance the prognosis of boar field fertility, the aim of this feasibility study was to record scrotal function and correlate the sperm variables with the frequency/intensity of scrotal contractions (SC) throughout the semen collection.

## 2. Materials and Methods

All operations of the study were carried out in agreement with the guidelines for animal research (96385-19929/2020-Project Number: HFRIFM17-2040). The used reagents were purchased from Sigma-Aldrich (Seelze, Germany) unless otherwise specified.

### 2.1. Experimental Design, Animals, Semen Collection and Processing

Five adult (12–20 months old) boars [Pietrain (3), Landrace (1) or crossbred Pietrain × Duroc (1)] of proven fertility, based on previous outcome of artificial inseminations, were involved in this research. Boars were individually housed under controlled conditions in a pig farm, and they were then fed with commercial boar diet, having an ad libitum intake of water. Two ejaculates per boar, meaning ten ejaculates in total, were collected bi-weekly, and these were extended by an MRA^®^ Extender (KUBUS, Madrid, Spain) to a final concentration of 30 × 10^6^ spermatozoa/mL. The prepared semen doses were used in the farm for the fertilization of the sows by AI, while one of them was transported to the laboratory for further analysis (transportation time about 45 min).

### 2.2. Visual Video Monitoring, Recording, and Processing

**Figure 1:**
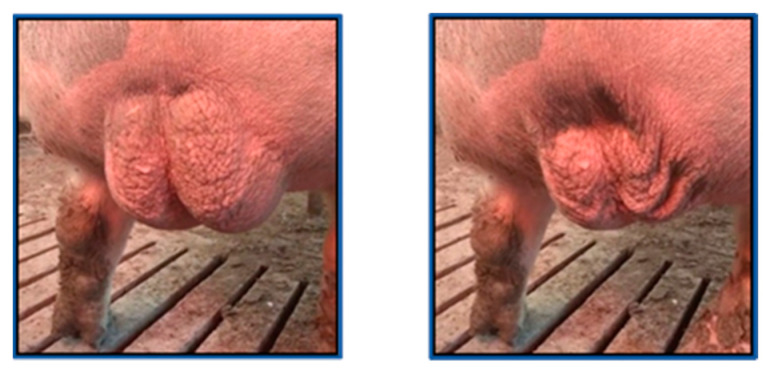
Video frames with relaxation (**left**) and contraction (**right**) of the boar scrotum.

The total ejaculation time was recorded by a timer. Ten (10) videos were recorded (two from each of the five boars). The video camera of a smartphone (Redmi Note 4X, Xiaomi) was placed at a constant position throughout the sperm collection process, viewing the rear part of the boar body. This section of the boar does not move significantly, ensuring an almost constant view angle of the boar scrotum. The camera was placed at about the same horizontal plane as that of the scrotum mid-plane. The further video processing was carried out by the Image Processing Toolbox of Matlab (Mathworks Inc., Natick, MA, USA), (Figure 1).

**Figure 2:**
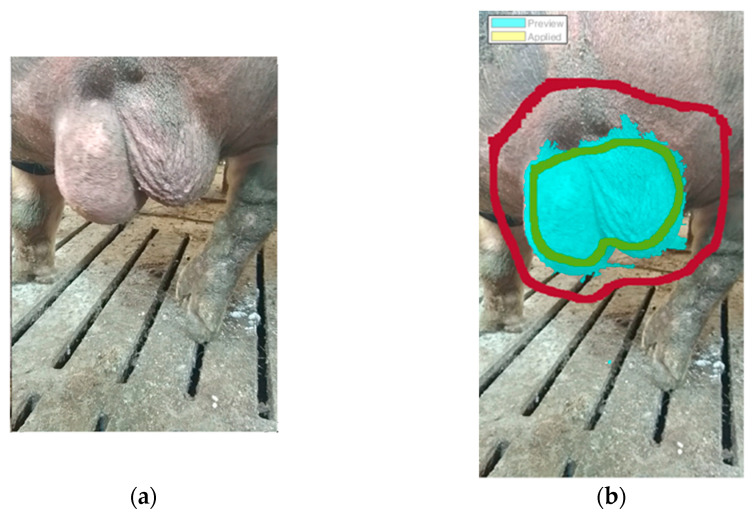
Segmentation of the scrotum area in a tagged video frame. (**a**) Unprocessed video frame imported in the Image Segmenter. (**b**) Video frame segmented with the Graph Cut algorithm, showing the foreground scribble (green color), the background scribble (dark red color) and the segmented area (cyan).

**Figure 3:**
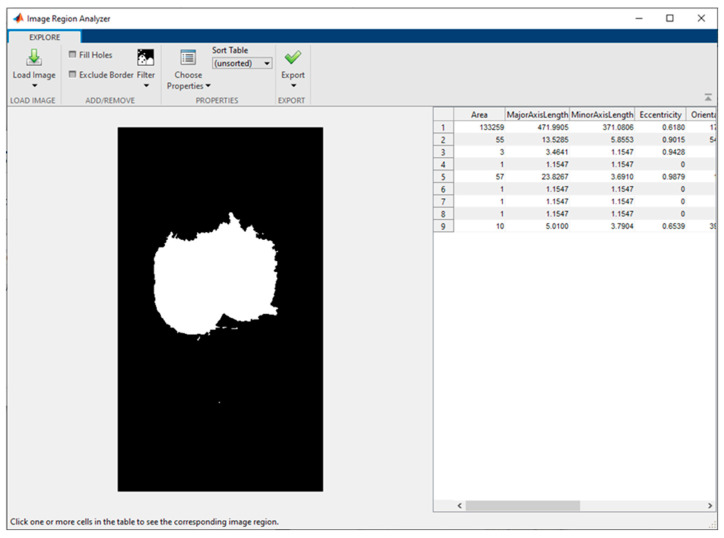
Analysis of the features of the segmented image of the area of interest.

The size of the scrotum was measured as a percentage of pixels of the total picture. The SC intensity was determined as a percentage change in the scrotum volume among the video frames of maximum contraction and relaxation, following these steps:

1. Load and view the video of the process in Movie Player. Tag the frames of maximum contraction (e.g., Figure 2a) and maximum relaxation of the scrotum.
2. For each frame of interest (contraction/relaxation), export the frame to the Image Tool and, subsequently, to Matlab’s Workspace.
3. Load the image from the Workspace into the Image Segmenter and use the Graph Cut algorithm: Mark the foreground with a scribble (green) and the background with another scribble (dark red); the region of interest (scrotum) is segmented (cyan pixels, Figure 2b).
4. Manipulate the region (fill holes, clear borders, erode mask) to make its boundary smoother. Create a mask (i.e., a black-and-white image) from the region (marked with a yellow color) and export it to Matlab’s Workspace.
5. Load the mask (black-and-white image) from the Workspace into the Image Region Analyzer to determine the number of pixels belonging to the area of the mask (the first line in Table with the regions’ properties, Figure 3).
6. Divide the number of pixels in the mask by the total number of image pixels to give a percentage. This is to be used as a proxy of the scrotum size (volume) in the video frame.

The intensity of each SC was evaluated as the percentage difference between the scrotum size in the frame of full contraction and the scrotum size in the frame of full relaxation, both sizes having been assessed in their respective video frames with the above steps as the occupied percentage of pixels in each video frame.

Since the way the scribbles are drawn affects image segmentation, the same operator was used for all frames and animals. Concerning the video processing time spending, each video frame tagging lasted 15 min and the image analysis 10 min maximum. Thus, 25–30 min was enough to obtain the video recording result of one ejaculation or to examine one boar under the semen collection process.

### 2.3. Semen Assessment

The aliquots of the extended semen were assessed for sperm: (a) motility and kinematics by a computer-assisted sperm analyzer (CASA), (b) nuclear chromatin integrity by acridine orange, (c) viability and morphology via a eosin-nigrosin stain assay, and (d) biochemical activity of cell membrane with a HOS-Test.

#### 2.3.1. Computer-Assisted Semen Analysis (CASA Analysis)

Semen samples (10 μL) were loaded in a preheated (37 °C) Makler^®^ champer and analyzed by computer-assisted semen analysis (CASA-Sperm Class Analyzer^®^, Microptic S.L., Automatic Diagnostic Systems, Barcelona, Spain) for motility and kinematic parameters [total/progressive motility %, rapid/medium/slow movement spermatozoa %, VCL—curvilinear velocity (μm/s), VSL—straight line velocity (μm/s), VAP—average path velocity (μm/s), ALH—amplitude of lateral head displacement (μm), BCF—beat/cross-frequency (Hz), LIN—linearity (VSL/VCL × 100), STR—straightness (VSL/VAP × 100), WOB—wobble (VAP/VCL × 100)]. The analysis configurations were as follows: 8 fields, at least 500 spermatozoa were recorded (×100), region of particle control 10–18 microns, progressive movement of >45% of the parameter STR, circumferential movement < 50% LIN, depth of field 10, temperature of the microscope plate 37 °C.

#### 2.3.2. Viability and Morphology

The Eosin-nigrosin stain was used to evaluate viability (%) and morphology (%), as is described in the laboratory manual of the World Health Organization (WHO) [12]. 

#### 2.3.3. Sperm Membrane Biochemical Activity

A hypo-osmotic swelling test (HOS-Test) was performed as described by Michos et al. [2]. In total, 200 spermatozoa per slide were evaluated (×400). The results were expressed as the percentage of spermatozoa with swollen tails.

#### 2.3.4. Sperm DNA Fragmentation

The acridine orange test (AOT) was performed for the assessment of the sperm DNA integrity (%), according to Tsakmakidis et al. [13].

### 2.4. Statistical Analysis

All measurements were analyzed with Matlab (Statistics and Machine Learning Toolbox) to find statistical correlations. More specifically, a linear mixed-effects model was used, assuming a fixed effect between any two variables, one of which was the predictor and the second of which was the response variable. It was further assumed that random effects for the intercept and the predictor variable were not correlated. For each linear model the grouping variable was the boar ID, since two measurements (ejaculations) were performed per boar. Each model resulted in a value for the linear regression coefficient, as well as the 95% confidence interval (CI) for this coefficient. Finally, the coefficient of determination (R^2^) was evaluated for each model. Statistically significant difference was defined as *p* < 0.05.

## 3. Results

**Figure 4:**
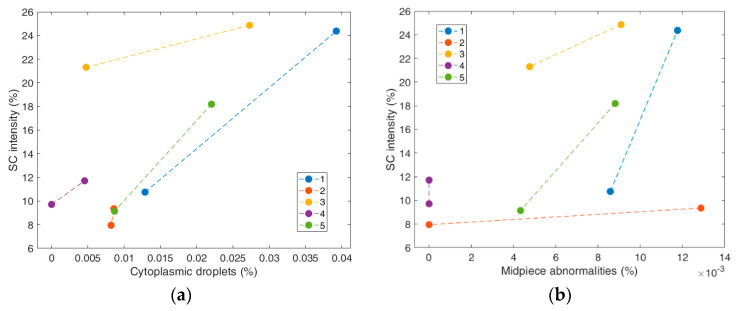
Plot of SC intensity (%) in relation to (**a**) cytoplasmic droplets (%), and (**b**) midpiece abnormalities (%), grouped by boar. SC intensity shows a significant and strong correlation with cytoplasmic droplets, but not with midpiece abnormalities. Each dot with a different color indicates a different boar.

The correlations among semen assessed parameters, total ejaculation time and scrotal volume are listed in Table 1. The results revealed significant and strong correlations (R^2^ > 0.5, *p* ≤ 0.05) of the SC intensity as a change in the scrotum volume in pixels, with the average path velocity VAP (R^2^ = 0.591, *p* = 0.043) and with the percentage of the cytoplasmic droplets (R^2^ = 0.509, *p* = 0.036). None of the examined semen samples were detected with sperm chromatin DNA fragmentation, since values ranged between 0–1%. For that reason, this variable was not correlated with the SC features. Figure 4 shows the plot of SC intensity as a relation of two morphological variables, i.e., cytoplasmic droplets, with which it has a significant and strong correlation, and midpiece abnormalities, with which it is not correlated.

## 4. Discussion

The extended use of boar semen for fertilizing purposes requires the performance of routine laboratory evaluations, including VAP, morphology and semen parameters, the latter of which were correlated with SC intensity in the present study. The cytoplasmic droplet is a small remaining part of cytoplasm which, during epididymal maturation, normally migrates from the neck to the tail of spermatozoa, up to its release. In fact, it is an essential energy source serving the epididymal sperm maturation but must be later removed [14]. Especially in boars, the cytoplasmic droplets are detached from spermatozoa within 1 min after ejaculation [15]. A high number of spermatozoa with cytoplasmic droplets in the ejaculate indicates poor management. Like the high frequency of semen collections, this is reflected in detrimental effects on the sperm maturation process. A common practice is to perform AI with boar ejaculates that fulfil the criteria of <20% morphologically abnormal sperm and <15% cytoplasmic droplets [16]. This is necessary, because it is well known that high percentages of cytoplasmic droplets are associated with subfertility cases and poor results of field fertility. This rule was supported by the findings of Waberski et al. [17], who reported a negative correlation between the percentage of spermatozoa with distal cytoplasmic droplets and both pregnancy rate and litter size. In addition, Lovercamp et al. [18] suggested that remaining cytoplasmic droplets do not affect the total number born piglets, but that they negatively influence the farrowing rate. Moreover, a human study reported that cytoplasmic droplets are not harmful to sperm motility, but that they are related to physiological semen volume regulation [19]. The results of the present study did not reveal a strong correlation of SC with sperm motility and kinematics, except with the VAP variable, where significant correlation was noticed. VAP is considered as the time-averaged velocity of the sperm head along its average path, indicating the forward speed of a spermatozoon [20]. It is an important parameter which has been significantly negatively correlated with seminal plasma thiobarbituric acid-reactive substances (TBARS) and intracellular superoxide (O_2_^−•^) levels in human sperm [21]. This means that VAP could be a critical sperm movement parameter when lipid peroxidation deleteriously affects the fertilization process. On the other hand, in pigs, previous studies characterized VAP as a parameter with a prognostic value of sperm fertilizing ability because of its positive correlation with fertility and live born piglets [22,23]. Additionally, a significant predictive capacity of VAP for litter size was also reported when the effects of sperm clusters on the fertility capacity of the boar ejaculate were investigated [23]. The results of the present study, where recordings of the scrotal function were carried out, revealed correlations of the intensity of SC with sperm variables with prognostic value. However, they are not enough to establish a new autonomous and sufficient prognostic model.

## 5. Conclusions

In conclusion, although boar scrotal contractions/relaxations monitoring at the time of semen collection has encouraging results with predictable values of some semen quality variables, more research is needed to establish its appropriateness as an accompanying method for the prognosis of boars’ ability to produce high-quality semen.

## Figures and Tables

**Table 1:** Correlations between sperm variables, scrotal volume and duration of the ejaculation.

Variables		Linear Regression Coefficient	95% Confidence Interval		*p* Value (*n* = 10)	R^2^
			Lower Coefficient	Upper Coefficient		
Total motility (%)	Change in the scrotum volume (%)	−0.157	−0.509	0.195	0.333	0.017
Progressive motility (%)	Change in the scrotum volume (%)	−0.671	−2.132	0.789	0.320	0.109
Non-progressive motility (%)	Change in the scrotum volume (%)	0.112	−1.272	1.049	0.829	0.643
Immotile spermatozoa (%)	Change in the scrotum volume (%)	0.157	−0.195	0.509	0.333	0.017
Rapid spermatozoa (%)	Change in the scrotum volume (%)	−0.279	−2.090	1.531	0.731	0.539
Medium spermatozoa (%)	Change in the scrotum volume (%)	−0.105	−0.950	0.741	0.783	0.328
Slow spermatozoa (%)	Change in the scrotum volume (%)	0.822	−0.241	1.886	0.112	0.147
VCL (μm/s)	Change in the scrotum volume (%)	−0.202	−1.287	0.883	0.679	0.784
VSL (μm/s)	Change in the scrotum volume (%)	0.154	−0.715	1.124	0.693	0.807
VAP (μm/s)	Change in the scrotum volume (%)	0.574	0.024	1.024	0.043	0.591
LIN (%)	Change in the scrotum volume (%)	0.939	0.175	1.702	0.022	0.337
STR (%)	Change in the scrotum volume (%)	1.111	0.315	1.906	0.012	0.448
WOB (%)	Change in the scrotum volume (%)	0.538	0.068	1.009	0.030	0.337
ALH (μm)	Change in the scrotum volume (%)	−0.029	−0.089	0.031	0.269	0.565
BCF (Hz)	Change in the scrotum volume (%)	0.055	−0.025	1.134	0.150	0.983
Normal morphology (%)	Change in the scrotum volume (%)	−0.004	0.007	−0.001	0.029	0.342
Abnormal morphology (%)	Change in the scrotum volume (%)	0.004	0.001	0.007	0.029	0.342
Head abnormalities (%)	Change in the scrotum volume (%)	0.002	−0.001	0.004	0.135	0.221
Midpiece abnormalities (%)	Change in the scrotum volume (%)	0.001	−0.001	0.001	0.184	0.071
Tail abnormalities (%)	Change in the scrotum volume (%)	0.001	−0.001	0.002	0.123	0.642
Cytoplasmic droplets (%)	Change in the scrotum volume (%)	0.001	0.001	0.002	0.036	0.509
Viability (%)	Change in the scrotum volume (%)	−0.004	0.009	0.001	0.078	0.201
Hyperactivated spermatozoa (%)	Change in the scrotum volume (%)	−0.125	0.269	0.017	0.077	0.353
Host (+) spermatozoa (%)	Change in the scrotum volume (%)	−0.003	−0.006	−0.001	0.133	0.121
Volume (mL)	Change in the scrotum volume (%)	−1.179	−10.115	7.756	0.769	0.912
Total ejaculation time (min)	Change in the scrotum volume (%)	4.404	0.801	8.007	0.022	0.373

Slow: spermatozoa with rapid, medium and slow movement, respectively, %; VCL: curvilinear velocity (μm/s); VSL: straight line velocity (μm/s); VAP: average path velocity (μm/sec); LIN: linearity (VSL/VCL × 100); STR: straightness (VSL/VAP × 100); WOB: wobble (VAP/VCL × 100); ALH: amplitude of lateral head displacement (μm); BCF: beat/cross-frequency (Hz); Volume: ejaculate volume (mL).

## Data Availability

The data are not publicly available because of to the project’s privacy restrictions, but they can be available on request to the corresponding author.
